# Supplementary material for: Combination of Two Long-Acting Antipsychotics in Schizophrenia Spectrum Disorders: A Systematic Review
Source: Brain Sci. 2024 Apr 26;14(5):433. doi: 10.3390/brainsci14050433 (PMC11117856; doi:10.3390/brainsci14050433)
Supplement: Supplementary file 1 [file brainsci-14-00433-s001.zip › Supplementary/STable 1_Key search dual LAI_070424.docx]

| **Supplementary Table 1**  Search key used for the extensive literature search for relevant articles on three different online databases | |
| --- | --- |
| **Online database** | **Key search** |
| ***PubMed*** | ((((("Drug Therapy, Combination"[Mesh]) OR ("combination") OR ("combining") OR ("concurrent")) AND ((("Antipsychotic Agents"[Mesh]) AND (("Delayed-Action Prepara-tions"[Mesh]) OR "depot")) OR (("Antipsychotic Agents"[Mesh]) AND (("Injections"[Mesh]) OR (injectable)) AND (("long-acting") OR ("long acting"))) OR ((LAI) OR (LAIs)) OR (LAIA) OR (LAIAs) OR ("long-acting injectable antipsychotic"))) OR ("dual LAIs") OR ("two LAIs") AND ("Schizophrenia"[Mesh]))) OR (("two long-acting"[title])) |
| ***Scopus*** | TITLE-ABS-KEY (((((( "combination" ) OR ( "combining" ) OR ("concurrent")) AND ((("Antipsychotic Agents") AND (("Delayed-Action Preparations") OR ("depot")) OR (("Antipsychotic Agents") AND (("Injections") OR (injectable)) AND (("long-acting") OR ("long acting"))) OR ((LAI) OR (LAIs) ) OR (LAIA) OR (LAIAs) OR ("long-acting injectable antipsychotic"))) OR ("dual LAIs") OR ("two LAIs") AND ("Schizophrenia"))) OR (("two long-acting"))) AND (LIMIT-TO (DOCTYPE, "ar")) AND (LIMIT-TO (LANGUAGE, "English" )) AND (LIMIT-TO (EXACTKEYWORD, "Human")) |
| ***APA PsycInfo*** | tiab (((((("combination") OR ("combining" ) OR ("concurrent")) AND ((("Antipsychotic Agents") AND (("Delayed-Action Preparations") OR "depot")) OR (("Antipsychotic Agents") AND (("Injections") OR (injectable)) AND (("long-acting") OR ("long acting"))) OR ((LAI) OR (LAIs) ) OR (LAIA) OR (LAIAs) OR ("long-acting injectable antipsychotic"))) OR ("dual LAIs") OR ("two LAIs") AND ("Schizophrenia"))) OR (("two long-acting")) |
|  | |
